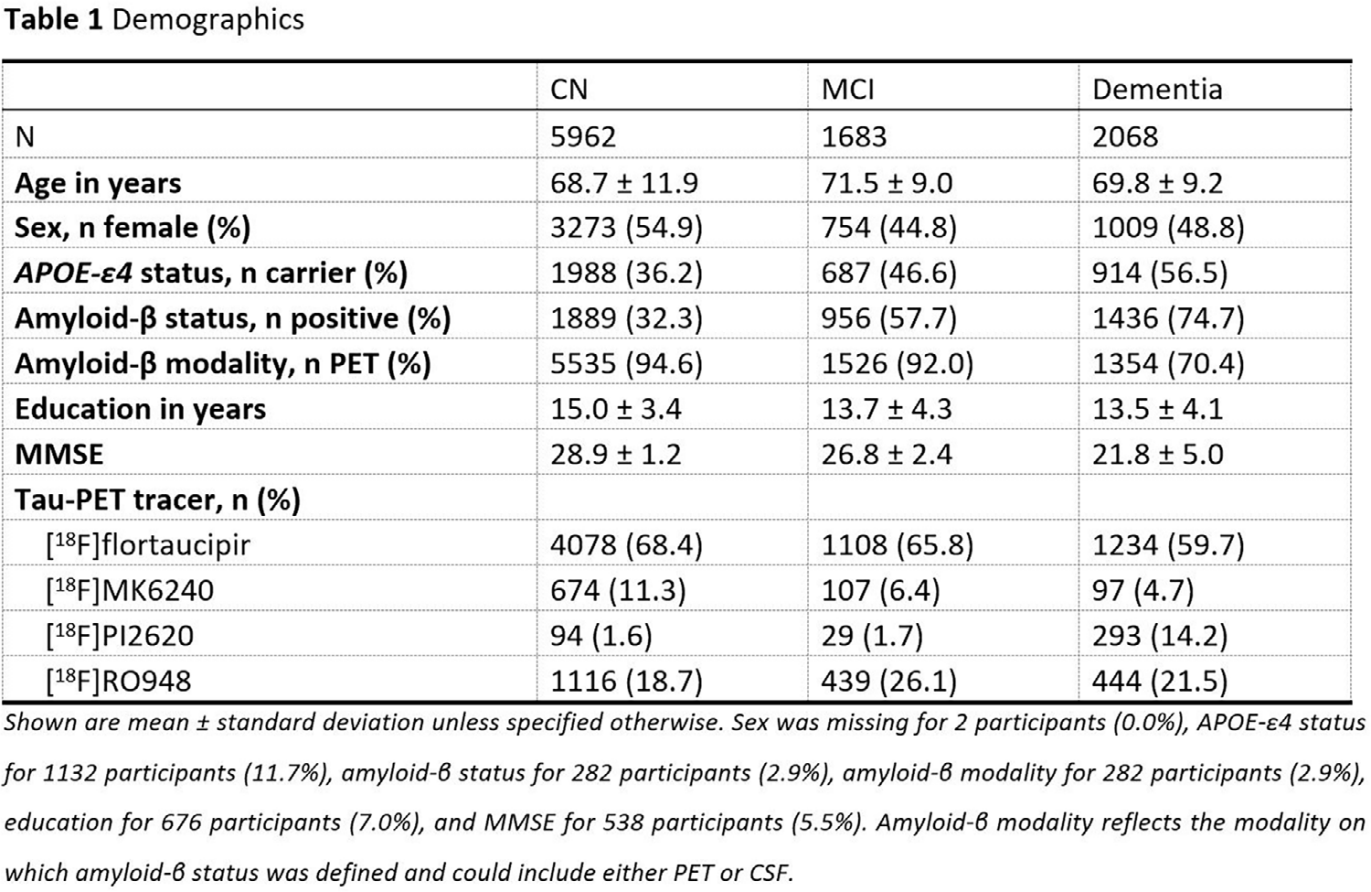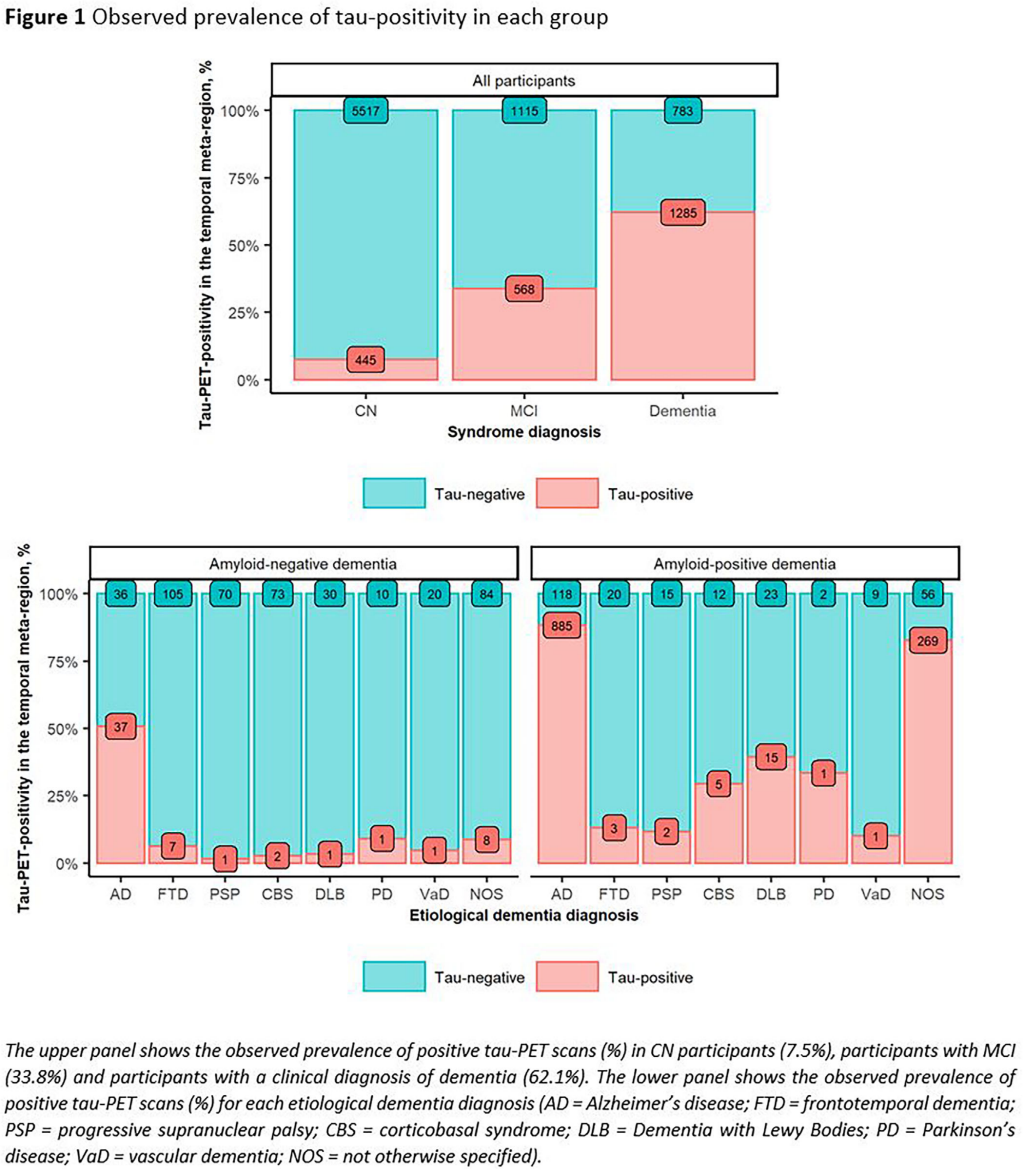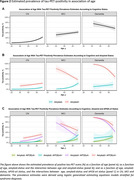# The prevalence of tau‐PET positivity in aging and dementia

**DOI:** 10.1002/alz.093782

**Published:** 2025-01-09

**Authors:** Emma M. Coomans, Colin Groot, Christopher C. Rowe, Vincent Dore, Victor L Villemagne, Elsmarieke van de Giessen, Wiesje M. van der Flier, Yolande A.L. Pijnenburg, Pieter Jelle Visser, Anouk den Braber, Michael Pontecorvo, Sergey Shcherbinin, Ian A. Kennedy, William J. Jagust, Suzanne L. Baker, Theresa M. Harrison, Juan Domingo Gispert, Mahnaz Shekari, Carolina Minguillon, Ruben Smith, Niklas Mattsson‐Carlgren, Sebastian Palmqvist, Olof Strandberg, Erik Stomrud, Maura Malpetti, John T O'Brien, James B Rowe, Elena Jäger, Gérard N Bischof, Alexander Drzezga, Valentina Garibotto, Giovanni Frisoni, Débora Elisa Peretti, Michael Schöll, Ingmar Skoog, Silke Kern, Reisa A Sperling, Keith A Johnson, Shannon L. Risacher, Andrew J. Saykin, Maria C. Carrillo, Brad C Dickerson, Liana G. Apostolova, Henryk Barthel, Michael Rullmann, Konstantin Messerschmidt, Rik Vandenberghe, Koen Van Laere, Laure Spruyt, Nicolai Franzmeier, Matthias Brendel, Johannes Gnörich, Tammie L.S. Benzinger, Julien Lagarde, Marie Sarazin, Michel Bottlaender, Sylvia Villeneuve, Judes Poirier, Sang Won Seo, Yuna Gu, Jun Pyo Kim, Elizabeth Mormino, Christina B. Young, Hillary Vossler, Pedro Rosa‐Neto, Joseph Therriault, Nesrine Rahmouni, William Coath, David M Cash, Jonathan M Schott, Gil D. Rabinovici, Renaud La Joie, Howard J. Rosen, Sterling C. Johnson, Bradley T. Christian, Tobey J. Betthauser, Oskar Hansson, Rik Ossenkoppele

**Affiliations:** ^1^ Alzheimer Center Amsterdam, Amsterdam UMC, Amsterdam Netherlands; ^2^ Alzheimer Center Amsterdam, Neurology, Vrije Universiteit Amsterdam, Amsterdam UMC location VUmc, Amsterdam Netherlands; ^3^ Molecular Research and Therapy, Austin Health and University of Melbourne, Heidelberg, VIC Australia; ^4^ Department of Molecular Imaging & Therapy, Austin Health, Heidelberg, VIC Australia; ^5^ University of Pittsburgh, Pittsburgh, PA USA; ^6^ Radiology & Nuclear Medicine, Vrije Universiteit Amsterdam, Amsterdam UMC location VUmc, Amsterdam Netherlands; ^7^ Alzheimer Center Amsterdam, Neurology, Vrije Universiteit Amsterdam, Amsterdam UMC, Amsterdam Netherlands; ^8^ Alzheimer Center Limburg, School for Mental Health and Neuroscience, Maastricht University, Maastricht Netherlands; ^9^ Eli Lilly and Company, Indianapolis, IN USA; ^10^ University of California, Berkeley, Berkeley, CA USA; ^11^ Lawrence Berkeley National Laboratory, Berkeley, CA USA; ^12^ Barcelona?eta Brain Research Center (BBRC), Pasqual Maragall Foundation, Barcelona Spain; ^13^ Clinical Memory Research Unit, Department of Clinical Sciences Malmö, Faculty of Medicine, Lund University, Lund Sweden; ^14^ Clinical Memory Research Unit, Department of Clinical Sciences, Lund University, Lund Sweden; ^15^ Department of Clinical Neurosciences and Cambridge University Hospitals NHS Trust, University of Cambridge, Cambridge United Kingdom; ^16^ Department of Psychiatry, University of Cambridge, Cambridge United Kingdom; ^17^ Department of Clinical Neurosciences, University of Cambridge, Cambridge United Kingdom; ^18^ University of Cologne, Faculty of Medicine and University Hospital Cologne, Department of Nuclear Medicine, Cologne Germany; ^19^ University of Cologne, Faculty of Medicine and University Hospital Cologne, Cologne Germany; ^20^ Division of Nuclear Medicine and Molecular Imaging, Geneva University Hospitals, Geneva Switzerland; ^21^ University Hospitals and University of Geneva, Geneva Switzerland; ^22^ Laboratory of Neuroimaging and Innovative Molecular Tracers (NIMTlab), University of Geneva, Neurocentre and Faculty of Medicine, Geneva Switzerland; ^23^ Wallenberg Centre for Molecular and Translational Medicine, University of Gothenburg, Gothenburg Sweden; ^24^ Neuropsychiatric Epidemiology, Institute of Neuroscience and Physiology, Sahlgrenska Academy, Centre for Ageing and Health (AGECAP) at the University of Gothenburg, Gothenburg Sweden; ^25^ Harvard Medical School, Cambridge, MA USA; ^26^ Massachusetts General Hospital, Department of Neurology, Harvard Medical School, Boston, MA USA; ^27^ Center for Neuroimaging, Department of Radiology and Imaging Sciences, Indiana University School of Medicine, Indianapolis, IN USA; ^28^ Indiana University School of Medicine, Indianapolis, IN USA; ^29^ Alzheimer’s Association, Chicago, IL USA; ^30^ Massachusetts General Hospital, Boston, MA USA; ^31^ Leipzig University Medical Center, Leipzig Germany; ^32^ Department of Nuclear Medicine, University of Leipzig, Leipzig Germany; ^33^ University Hospital Leuven, Leuven Belgium; ^34^ KU Leuven and University Hospital Leuven, Leuven Belgium; ^35^ Laboratory for Cognitive Neurology, KU Leuven, Leuven Belgium; ^36^ Institute for Stroke and Dementia Research (ISD), University Hospital, LMU, Munich, Bayern Germany; ^37^ Department of Nuclear Medicine, University Hospital, LMU Klinikum, Munich, Bavaria Germany; ^38^ University Hospital of Munich, Munich Germany; ^39^ Mallinckrodt Institute of Radiology, Washington University in St. Louis, St. Louis, MO USA; ^40^ Unit of Neurology of Memory and Language, Université Paris Cité, GHU Paris Psychiatry and Neurosciences, Hôpital Sainte Anne, Paris France; ^41^ Neurology of Memory and Language Department, GHU Paris Psychiatrie & Neurosciences, Hôpital Sainte‐Anne, Paris France; ^42^ Université Paris‐Saclay, CEA, CNRS, Inserm, BioMaps, Orsay France; ^43^ Douglas Mental Health Research Centre, Montreal, QC Canada; ^44^ Department of Medicine, McGill University, Montréal, QC Canada; ^45^ Alzheimer’s Disease Convergence Research Center, Samsung Medical Center, Seoul Korea, Republic of (South); ^46^ Samsung Medical Center, Sungkyunkwan University School of Medicine, Gangnam‐gu, Seoul Korea, Republic of (South); ^47^ Department of Neurology and Neurological Sciences, Stanford University School of Medicine, Stanford, CA USA; ^48^ Stanford University School of Medicine, Stanford, CA USA; ^49^ Translational Neuroimaging Laboratory, The McGill University Research Centre for Studies in Aging, Montréal, QC Canada; ^50^ Translational Neuroimaging Laboratory, The McGill University Research Centre for Studies in Aging, Montreal, QC Canada; ^51^ Dementia Research Centre, UCL Queen Square Institute of Neurology, University College London, London United Kingdom; ^52^ Dementia Research Centre, UCL Queen Square Institute of Neurology, London United Kingdom; ^53^ Weill Institute for Neurosciences, University of California, San Francisco, San Francisco, CA USA; ^54^ University of California, San Francisco, San Francisco, CA USA; ^55^ Memory and Aging Center, UCSF Weill Institute for Neurosciences, San Francisco, CA USA; ^56^ Wisconsin Alzheimer’s Disease Research Center, University of Wisconsin‐Madison School of Medicine and Public Health, Madison, WI USA; ^57^ University of Wisconsin‐Madison, Madison, WI USA; ^58^ Alzheimer’s Disease Research Center, University of Wisconsin‐Madison, Madison, WI USA; ^59^ Clinical Memory Research Unit, Department of Clinical Sciences, Lund University, and Memory Clinic, Skåne University Hospital, Malmö Sweden

## Abstract

**Background:**

Tau‐PET imaging allows in‐vivo detection of neurofibrillary tangles. One tau‐PET tracer (i.e., [18F]flortaucipir) has received FDA‐approval for clinical use, and multiple other tau‐PET tracers have been implemented into clinical trials for participant selection and/or as a primary or secondary outcome measure. To optimize future use of tau‐PET, it is essential to understand how demographic, clinical and genetic factors affect tau‐PET‐positivity rates.

**Method:**

This large‐scale multi‐center study includes 9713 participants from 35 cohorts worldwide who underwent tau‐PET with [18F]flortaucipir (n = 6420), [18F]RO948 (n = 1999), [18F]MK6240 (n = 878) or [18F]PI2620 (n = 416) (Table‐1). We analyzed individual‐level tau‐PET SUVR data using a cerebellar reference region that were processed either centrally (n = 3855) or by each cohort (n = 5858). We computed cohort‐specific SUVR thresholds based on the mean + 2 standard deviations in a temporal meta‐region of amyloid‐negative cognitively normal (CN) individuals aged >50. Logistic generalized estimating equations were used to estimate tau‐PET‐positivity probabilities, using an exchangeable correlation structure to account for within‐cohort correlations. Analyses were performed with (interactions between) age, amyloid‐status, and APOE‐e4 carriership as independent variables, stratified for syndrome diagnosis.

**Result:**

The study included 5962 CN participants (7.5% tau‐PET‐positive), 1683 participants with mild cognitive impairment (MCI, 33.8% tau‐PET‐positive) and 2068 participants with a clinical diagnosis of dementia (62.1% tau‐PET‐positive) (Figure‐1). From age 60 to 80 years, the estimated prevalence of tau‐PET‐positivity increased from 1.2% [95% CI: 0.9%‐1.5%] to 3.7% [2.3%‐5.1%] among CN amyloid‐negative participants; and from 16.4% [10.8%‐22.1%] to 20.5% [18.8%‐22.2%] among CN amyloid‐positive participants. Among amyloid‐negative participants with MCI and dementia, from age 60 to 80 years, the estimated prevalence of tau‐PET‐positivity increased from 3.5% [1.6%‐5.3%] to 11.8% [7.1%‐16.5%] and from 12.6% [4.5%‐20.7%] to 15.9% [6.7%‐25.1%] respectively. In contrast, among amyloid‐positive participants with MCI and dementia, from age 60 to 80 years, the estimated prevalence of tau‐PET‐positivity decreased from 66.5% [57.0%‐76.0%] to 48.3% [42.9%‐53.8%] and from 92.3% [88.7%‐95.9%] to 73.4% [67.5%‐79.3%] respectively. APOE‐e4 status primarily modulated the association of age with tau‐PET‐positivity estimates among CN and MCI amyloid‐positive participants (Figure‐2).

**Conclusion:**

This large‐scale multi‐cohort study provides robust prevalence estimates of tau‐PET‐positivity, which can aid the interpretation of tau‐PET in the clinic and inform clinical trial designs.